# Histone Deacetylase Inhibitors Impair Glioblastoma Cell Motility and Proliferation

**DOI:** 10.3390/cancers14081897

**Published:** 2022-04-09

**Authors:** Elena Rampazzo, Lorenzo Manfreda, Silvia Bresolin, Alice Cani, Elena Mariotto, Roberta Bortolozzi, Alessandro Della Puppa, Giampietro Viola, Luca Persano

**Affiliations:** 1 Department of Women and Children’s Health, University of Padova, Via Giustiniani 3, 35128 Padova, Italy; lorenzo.manfreda@phd.unipd.it (L.M.); silvia.bresolin@unipd.it (S.B.); cnalca@unife.it (A.C.); elena.mariotto@unipd.it (E.M.); giampietro.viola.1@unipd.it (G.V.); luca.persano@unipd.it (L.P.); 2 Pediatric Research Institute, Corso Stati Uniti 4, 35127 Padova, Italy; roberta.bortolozzi@unipd.it; 3 Neurosurgery Unit, University Hospital of Padova, Via Giustiniani 2, 35128 Padova, Italy; alessandro.dellapuppa@unifi.it

**Keywords:** glioblastoma, histone deacetylase inhibitors, Wnt signaling, cell migration, interferon pathway

## Abstract

**Simple Summary:**

Glioblastoma (GBM) is considered the deadliest brain tumor; with patients displaying a high incidence of relapse and a 3-year survival of only 3–5%. For these reasons, investigation of the molecular basis of the disease could provide novel targets for therapy and improve patient prognoses. Based on our previous data, demonstrating that high levels of the transcription factor TCF4 (*TCF7L2*) sustain the aggressiveness and the stem cell features of these tumors, in this study we tested the ability of the histone deacetylase inhibitors (HDI) Trichostatin-A and Vorinostat to suppress TCF4 levels. We demonstrated that HDI treatment impairs proliferation and viability of GBM cells. Moreover, molecular analysis of HDI effects disclosed their ability to counteract tumor cell motility by affecting the RhoA-GTPase and the interferon pathways, supporting their further characterization as potential anti-GBM agents.

**Abstract:**

Despite being subjected to high-dose chemo and radiotherapy, glioblastoma (GBM) patients still encounter almost inevitable relapse, due to the capability of tumor cells to disseminate and invade normal brain tissues. Moreover, the presence of a cancer stem cell (CSC) subpopulation, already demonstrated to better resist and evade treatments, further frustrates potential therapeutic approaches. In this context, we previously demonstrated that GBM is characterized by a tightly-regulated balance between the β-catenin cofactors TCF1 and TCF4, with high levels of TCF4 responsible for sustaining CSC in these tumors; thus, supporting their aggressive features. Since histone deacetylase inhibitors (HDI) have been reported to strongly reduce TCF4 levels in colon cancer cells, we hypothesized that they could also exert a similar therapeutic action in GBM. Here, we treated primary GBM cultures with Trichostatin-A and Vorinostat, demonstrating their ability to strongly suppress the Wnt-dependent pathways; thus, promoting CSC differentiation and concomitantly impairing GBM cell viability and proliferation. More interestingly, analysis of their molecular effects suggested a prominent HDI action against GBM cell motility/migration, which we demonstrated to rely on the inhibition of the RhoA-GTPase and interferon intracellular cascades. Our results suggest HDI as potential therapeutic agents in GBM, through their action on multiple cancer hallmarks.

## 1. Introduction

Glioblastoma (GBM) is a devastating malignant brain tumor, whose management remains a big challenge for both clinicians and researchers. Despite, great advancements in the genomic and transcriptomic characterization of these tumors being achieved [1,2] in recent years, the therapeutic options for GBM patients have remained de facto unchanged for almost thirty years, being still based on maximal safe resection of the mass, followed by radiotherapy and adjuvant chemotherapy with alkylating agents such as Temozolomide (TMZ) and Carmustine [3,4]. Cell dissemination and the ability to invade normal brain parenchyma represent peculiar malignant features of GBM; making tumor relapse almost inevitable, due to the presence of quiescent residual cells endowed with stem cell characteristics that escape surgery [5,6]. These so-called cancer stem cells (CSC) display enhanced resistance to treatment and are considered the main drivers of tumor progression and relapse [7,8]. With the aim of characterizing the molecular basis of CSC maintenance and differentiation in GBM, we recently reported that this process is finely controlled by a molecular interplay occurring between the Wnt/β-catenin transcriptional co-factors TCFs and the Hypoxia Inducible Factor-1α (HIF-1α). In particular, we demonstrated that β-catenin and its co-factor TCF1 can interact with HIF-1α, in this way activating a transcriptional program that drives neuronal differentiation of CSC. However, high Molecular Weight (hMW) TCF4 isoforms concurrently exert an inhibitory function on Wnt signaling activation and GBM cell differentiation; thus, counteracting the formation of such a transcriptional complex, and eventually sustaining their aggressive, stem-like phenotype [9,10]. In line with our results, TCF4 has been described to act as a transcriptional repressor in various contexts, mainly depending on the recruitment of additional co-factors and peculiar histone modifiers [11,12]. Indeed, a previous study demonstrated that TCF4 transcriptional repression is dependent on intact histone deacetylase (HDAC) activity, in order to finely regulate neuronal fate specification in the vertebrate brain [13]. Accordingly, HDAC inhibition was reported to induce Wnt signaling activation and a concomitant potent neurogenic differentiation in adipose tissue-derived mesenchymal stem cells [14]. In this context, the HDAC inhibitors (HDI) of the hydroxamate class Trichostatin-A (TSA) and Vorinostat (suberoylanilide hydroxamic acid; SAHA) have been demonstrated to promote a proteasome-dependent depletion of TCF4 levels in colon cancer cells; thus, significantly affecting their proliferation and viability [15].

Based on these data, TCF4 may be considered a promising target for GBM treatment, to be therapeutically suppressed to ignite CSC differentiation, reduce cell malignancy, and even sensitize cancer cells to drugs. Moreover, based on the suggested interplay existing between TCF4 and HDACs in sustaining/suppressing peculiar transcriptional programs, HDAC inhibition could represent an effective strategy to impair the TCF4 repressive function and unlock the prodifferentiative potential of GBM CSCs, finally affecting tumor aggressiveness.

In this study, we treated patient derived GBM cultures with TSA and SAHA and demonstrated their ability to strongly reduce TCF4 levels, as previously shown in colon cancer [15]; thus, impairing cell stemness and even sensitizing them to the standard chemotherapeutic treatment with TMZ. However, HDAC inhibition was not accompanied by GBM differentiation to a specific cell lineage, due to an observed de-activation of Wnt signaling. Moreover, a deepened molecular analysis of the transcriptional effects exerted by HDI on GBM cells disclosed their ability to heavily impact cell proliferation, by affecting cell-cycle dynamics. Intriguingly, transcriptional data clearly suggested that HDI treatment was sufficient to inhibit a series of interferon pathway transcriptional targets. In this context, previous studies revealed the involvement of interferon (IFN) and other pro-inflammatory signals in sustaining the migratory and pro-invasive abilities of cancer cells, including glioma [16,17,18]. In particular, although interferon-stimulating genes (ISGs) have already been well studied for their antiviral properties; some of them share peculiar structural domains that make them particularly ‘interactive’ in the intracellular space and even able to stimulate epithelial to mesenchymal transition in solid tumors [19,20]. Accordingly, an increasing number of studies have utilized various ISGs as potential cancer prognostic markers endowed with oncogenic functions [21,22,23,24].

Here, we demonstrated that HDI significantly inhibit GBM cell motility/migration by affecting the expression of several IFN signaling target genes, which, if restored, are able to counteract HDI-dependent anti-migratory effects.

## 2. Materials and Methods

### 2.1. Cell Cultures

Primary GBM cells were isolated from GBM tumors at surgery (Supplementary Table S1) and cultured, as previously described [25]. Briefly, GBM samples were enzymatically and mechanically dissociated into single cell suspensions. Cells were then placed on fibronectin-coated plates and grown as monolayers in DMEM/F12 (Biowest, Nuaillé, France) supplemented with 10% BIT9500 (Stem Cell Technologies, Vancouver, BC, Canada), 20 ng/mL basic Fibroblast Growth Factor (bFGF), and 20 ng/mL Epidermal Growth Factor (EGF; both from Cell Guidance Systems Ltd., Cambridge, UK). GBM cells were maintained in an atmosphere of 2% oxygen, 5% carbon dioxide, and balanced nitrogen in a H35 hypoxic cabinet (Don Whitley Scientific Ltd., Shipley, UK), to better resemble the hypoxic conditions of the GBM microenvironment [8,25].

### 2.2. Western Blot

Equal amounts of proteins extracted from control and HDI-treated GBM cells (10 µg) were resolved using SDS-PAGE gels (NuPage; Thermo Fisher Scientific, Waltham, MA, USA) and transferred to a polyvinylidene difluoride (PVDF) Immobilon-p membrane (Merck-Millipore, Darmstadt, Germany). Membranes were saturated with I-block™ (Thermo Fisher Scientific, Waltham, MA, USA) for at least 1 h at room temperature and then incubated overnight at +4 °C under constant shaking, with the following primary antibodies: anti-GAPDH (1:1000, Santa Cruz Biotechnology, Dallas, TX, USA), anti-HIF-1α (1:500, BD Biosciences, Franklin Lakes, NJ, USA), anti-β-catenin (1:1000, Abcam, Cambridge, UK), anti-TCF1 (1:500), anti-TCF4 (1:1000) (both from Cell Signaling Technology Inc., Danvers, MA, USA), anti-MMP9 (1:500), anti-RhoA (1:1000), anti-Rock2 (1:500), anti-phospho-Cofilin (S3, 1:1000), and anti-total Cofilin (1:1000) (all from Santa Cruz Biotechnology, Dallas, TX, USA). Anti-Acetylated Tubulin (1:1000, Santa Cruz Biotechnology, Dallas, TX, USA) was used as positive control of HDI-mediated inhibition of protein deacetylation activity. Anti-β-actin (1:25,000, Sigma-Aldrich, St. Louis, MO, USA) and Coomassie staining (Thermo Fisher Scientific, Waltham, MA, USA) were used as loading controls. Membranes were next incubated with peroxidase-conjugated secondary antibodies (Perkin Elmer, Waltham, MA, USA) and visualized using ECL Select (Cytiva, Marlborough, MA, USA). Images were acquired using the iBright FL1500 Imaging System (Thermo Fisher Scientific, Waltham, MA, USA). Densitometric analysis was performed using ImageJ (https://imagej.nih.gov accessed on 31 January 2020). 

### 2.3. Flow Cytometry

The expression of CD133, Nestin, Sox2, and βIII-tubulin was measured by flow cytometry using a Human Neural Lineage Analysis Kit (BD Biosciences, Franklin Lakes, NJ, USA), according to the manufacturer’s instructions. Samples were analyzed with a CytoFLEX flow cytometer (Beckman Coulter, Brea, CA, USA).

Cell cycle analysis was achieved by propidium iodide (PI) staining, according to standard procedures. Briefly, cells were seeded in 6-well plates (range of 50,000–250,000 cells/well depending on the cell culture used) and, after 24 h, they were treated with TSA (1 μM) or SAHA (2 μM). After 48 h, cells were harvested and fixed in cold 70% ethanol for at least 2 h, permeabilized with 0.1% Triton X-100 (Sigma Aldrich, St. Louis, MO, USA), and then stained with a PI/RNAse A solution (Sigma Aldrich, St. Louis, MO, USA and Qiagen, Hilden, Germany, respectively), before being detected with a Cytomics FC500 cytometer (Beckman Coulter, Brea, CA, USA). Analysis was performed with MultiCycle Software (Phoenix Flow Systems, San Diego, CA, USA), and the results are presented as percentage of the cells in each phase of cell-cycle (G_0_/G_1_, S, and G_2_/M). In some experiments, additional phospho-Histone H3 (S10) staining (Biolegend Inc., San Diego, CA, USA) allowed further specification of the percentage of cells in the M phase.

To measure HDI-induced apoptosis/cell death, GBM cells were treated with TSA or SAHA, as indicated, and then stained with an Annexin-V Fluos Kit, according to manufacturer’s instructions (Roche Diagnostics, Rotkreuz, Switzerland). Samples were analyzed using a Cytomics FC500 flow cytometer (Beckman Coulter, Brea, CA, USA).

5-ethynil-2′-deoxyuridine (EdU) incorporation was performed according to the Baseclick EdU Flow Cytometry Kit instructions (Sigma Aldrich, St. Louis, MO, USA). In particular, GBM cells (HuTuP82 and 176) were treated with TSA (1 μM) or SAHA (2 μM) for 48 h and then 10 µM EdU was added to the culturing medium and allowed to be incorporated by cells during DNA replication for 16 h. EdU incorporation was detected with a Cytomics FC500 cytometer (Beckman Coulter, Brea, CA, USA) and analyzed with FlowJo_v10.8.0 software (BD Bioscience, Franklin Lakes, NJ, USA).

### 2.4. Limiting Dilution Assay

To assess GBM cell self-renewal, they were seeded in 6-well plates (range of 200,000–300,000 cells/well depending on the cell culture used), treated with TSA (5 µM) or SAHA (5 µM) for 24 h, and then re-plated using a MoFlo XDP cell sorter (Beckman Coulter, Brea, CA, USA), in serial dilutions ranging from 0 to 500 cells/well in ultra-low attachment 96-well plates (Corning, Glendale, AZ, USA). Cells were cultured for two additional weeks, and then the proportion (%) of wells in which sphere formation was not observed was calculated. Statistical significance was calculated by the extra sum-of-squares F test.

### 2.5. Cell Treatments

GBM cells were seeded in 96-well plates (range of 5000–20,000 cells/well depending on the cell culture used) and, after 24 h, treated with scalar doses of TSA, SAHA, or TMZ (Selleck Chemicals, Houston, TX, USA). Cell viability was measured using a resazurin-based assay at 72 h from treatment with a Spark 10 M fluorimeter (Tecan, Männedorf, Swtzerland). EC_50_ was defined as the compound concentration required to inhibit cell proliferation or reduce cell viability by 50%, relative to matched DMSO-treated cells. All treatments were performed at least in triplicate for each cell culture used.

In some experiments, the potential synergistic/antagonistic behavior of TSA or SAHA combined with TMZ was evaluated in GBM cells, according to the Bliss excess method [26]. Briefly, primary GBM cells were seeded in 384-well plates (4000 cells/well) and, after 24 h, treated with TSA or SAHA as single agents, or combined with TMZ by a 5 × 5 matrix design. After 72 h of treatment, cells were assayed using a resazurin-based cell viability test. To ensure treatment reproducibility, all procedures, including cell seeding, drug dilution, cell treatment, and application of resazurin solution were carried out with a 96-channel robotic liquid handler (Microlab STAR 96-CORE, Hamilton, Bonaduz, Switzerland). The Bliss excess was then computed for all technical replicates per dose per drug combination. Data were processed using R3.6.3 and Rstudio Version 1.3.1056, according to Flobak et al. [27].

### 2.6. Immunofluorescence of Cultured Cells

GBM cells were cultured on 4-well chamber slides (range of 5000–10,000 cells/well depending on the cell culture used; BD Bioscience, Franklin Lakes, NJ, USA), treated with TSA and SAHA as indicated, fixed in cold 4% formaldehyde, and then washed and stored at +4 °C in PBS prior to analysis. Nestin (1:200; Merck-Millipore, Darmstadt, Germany), βIII-tubulin (1:500; Biolegend Inc., San Diego, CA, USA), Ki67 (1:100; Agilent Technologies, Santa Clara, CA, USA) primary antibody or TRITC-phalloidin (50 μg/mL; Sigma-Aldrich, St. Louis, MO, USA) were incubated according to the manufacturer’s instructions. Cells were then washed and incubated with species-specific secondary antibodies conjugated to Alexa dyes (1:1000; Thermo Fisher Scientific, Waltham, MA, USA). Cells were counterstained with DAPI (1 μg/mL; Sigma-Aldrich, St. Louis, MO, USA). 

In some experiments, control and HDI treated cells were stained with DAPI and analyzed for the presence of mitotic nuclei. Mitotic index was calculated as the percentage of mitotic nuclei per analyzed field.

Stainings were visualized using a LSM800 confocal microscope or an Axio Imager M1 epifluorescence microscope (Zeiss, Jena, Germany).

### 2.7. GBM Cell Transfection and Luciferase Reporter Assays

GBM cells were transiently transfected using the TransIT^®^-LT1 Transfection Reagent (Mirus Bio LLC, Madison, WI, USA), according to manufacturer’s indications.

To evaluate the impact of HDI treatment on TCF4 over-expressing cells, in terms of stemness, GBM cells were transfected with equal amounts of pcDNA3.1-TCF4E plasmid (Addgene plasmid #32738) or the relative empty vector, and, after 24 h, they were treated with HDI (1 μM TSA and 2 μM SAHA) for 48 h, until processed for verification of TCF4 over-expression through Western blot, and evaluated for the expression of the stem cell marker CD133 using flow cytometry, as described.

For luciferase reporter assays, GBM cells (HuTuP15, HuTuP61, and HuTuP82) were transfected with a Wnt-activation luciferase reporter construct (Bat-lux; Addgene plasmid #20890. Bat-lux luciferase expression depends on seven TCF/LEF binding sites upstream of the *siamois* gene minimal TATA box; thus, providing a Wnt signaling activation-dependent firefly luciferase production [28]. Cells were transfected 24 h after seeding with both BAT-lux and an equal amount of the pMAX-GFP plasmid as a transfection efficiency reference. The day after, they were treated with TSA (5 μM) and SAHA (5 μM) and after additional 24 h lysed in CCL buffer. Upon addition of a D-luciferin firefly solution (Luciferase Assay System, Promega, Madison, WI, USA), luciferase activity was measured using a Spark 10M instrument (Tecan, Männedorf, Swtzerland). Values are expressed as relative light units (RLUs) after being doubly normalized according to efficiency of transfection (GFP fluorescence) and control cells.

### 2.8. Gene Expression Profiling and Data Analysis

RNA was extracted from GBM cells using QIAzol reagent (Qiagen, Hilden, Germany), according to the manufacturer’s instructions. For microarray experiments, in vitro transcription, hybridization, and biotin labeling of RNA were performed, with a WT GeneChip Clariom™ S assay (Affymetrix, Santa Clara, CA, USA). Genechips were scanned with an Affymetrix 7G scanner, and the generated CEL files were normalized with the robust multi-array averaging (RMA) algorithm in Affy-R package (www.r-project.com; accessed on 10 July 2020). Differentially expressed genes between Control and TSA, or Control and SAHA, (both compounds were used at 5 μM for 24 h in HuTuP61 and HuTuP176 GBM primary cultures) were identified using Significance Analysis of Microarray (100 permutations, FDR q-value < 0.05) [29]. Clustering analysis was performed using Euclidean distance and the Ward.D method.

Enrichment analyses were performed on common differentially expressed genes between the two different primary cultures (Supplementary Figure S4A), by applying over-representation tests to the C2cp, C2cgp, and Hallmarks MSig databases. The most significant enrichments (FDR q-value < 0.05) are reported. 

The single sample gene set enrichment analysis (ssGSEA) approach was applied, in order to search for single sample overlaps in predetermined datasets of interest (C2cgp). ssGSEA results are represented by a levelplot generated in Morpheus (https://software.broadinstitute.org/morpheus/; accessed on 23 June 2021), which shows the relative enrichment score of each sample in the indicated gene sets.

### 2.9. Reverse Transcription and Quantitative Real-Time (RT) PCR

Total RNA (1–2 μg) was reverse-transcribed using a SuperScript^TM^ First-Strand Synthesis System (Thermo Fisher Scientific, Waltham, MA, USA), according to the manufacturer’s instructions. Quantitative real-time PCR was performed using Platinum SYBR Green Q-PCR Super Mix (Thermo Fisher Scientific, Waltham, MA, USA) and analyzed on a 7900HT ABI PRISM instrument (Applied Biosystems, Foster City, CA, USA). Specificity of primers was initially verified using Primer-BLAST (https://www.ncbi.nlm.nih.gov/tools/primer-blast/ accessed on 10 July 2020) and then confirmed by analysis of the dissociation curves generated for each primer couple. The oligonucleotides used for PCR amplification are listed in Supplementary Table S4. Expression values were normalized to GUSB according to the ΔΔCt method. Data are reported as log_2_ fold change expression of indicated genes relative to control cells.

### 2.10. Migration/Motility Assays

Live motility experiments were performed in 12-well plates (range of 20,000–50,000 cells/well depending on the cell culture used), in which cells were treated with TSA (1 μM), SAHA (2 μM), IFN-γ (1 μg/mL), or their combination, the day after seeding. To measure cell migration, 6 different areas per well were randomly recorded overnight (~16 h), to achieve a time-lapse monitoring of cell movements with an Axio Observer microscope (Zeiss, Jena, Germany) with controlled atmosphere and temperature. Images were captured every 30 min during a 16 h timespan and then cellular motility was analyzed by tracing single cell coordinates (x, y) with the MTrackJ plugin of ImageJ (https://imagej.nih.gov; accessed on 31 January 2020).

To measure cell ability to close a wound during scratch assays, GBM cells (HuTuP61, HuTuP82 and HuTuP176) were plated onto 12-well plates at high confluence (100,000–150,000 cells/well depending on the primary culture), and, after 24 h, the cell monolayer was scratched and treated or not with HDI (1 μM TSA and 2 μM SAHA). Then, the ability of edge cells to move into and close the scratch during time (24 and 48 h) was measured. Images were acquired with a Nikon TS100 inverted microscope (Nikon, Melville, NY, USA) and wound width was measured in four random fields/well using Adobe Photoshop CS6 (Adobe Systems Incorporated, La Jolla, CA, USA).

### 2.11. Statistical Analysis

Data used for generating displayed graphs were analyzed using statistical tools provided within GraphPad Prism 8.0.1 software (GraphPad, La Jolla, CA, USA). Bar graphs display data arranged as mean ± standard error of the mean (S.E.M.). One-way ANOVA with Newman–Keuls multiple comparison post-test was used for comparing data from three or more experimental groups. In addition, we used paired t-test for comparing two groups. Asterisks indicate a statistically significant difference with control cells (over bars) or selected experimental groups (over brackets, when present). In particular, * *p* < 0.05, ** *p* < 0.01, *** *p* < 0.001, **** *p* < 0.0001.

## 3. Results

### 3.1. HDI TSA and SAHA Suppress TCF4 Levels and Affect GBM Cell Stemness

**Figure 1:**
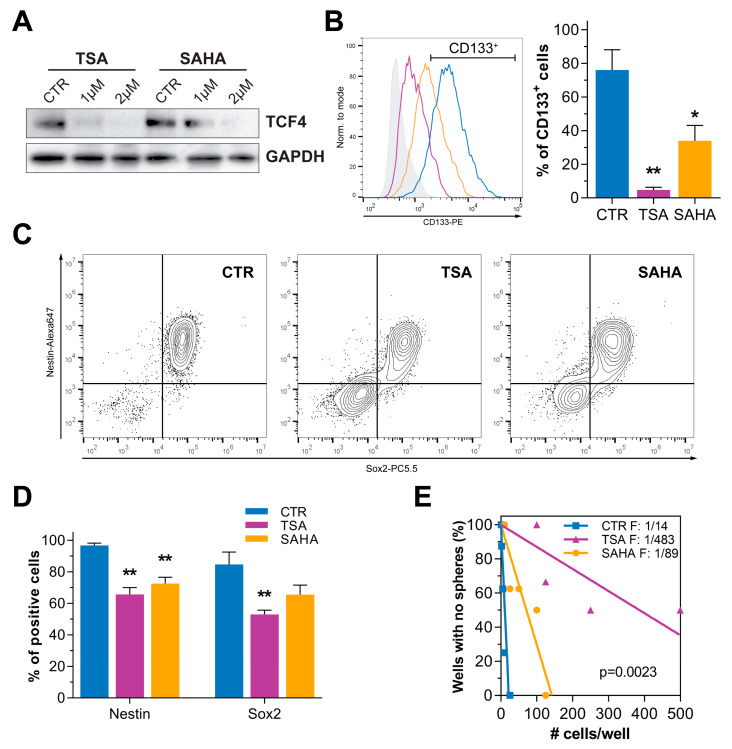
HDI treatment reduces TCF4 levels and impairs CSC phenotype and self-renewal. (**A**) Western blot analysis displaying TCF4 protein levels upon TSA and SAHA exposure (1–2 μM) for 24 h (HuTuP53). GAPDH was used as loading control. The original Western blot images included in (**A**) are provided in Supplementary Figure S1A. (**B**) Representative CD133 surface expression in control and TSA (1 μM)/SAHA (2 μM) treated GBM cells (72 h; HuTuP197) (left) and graph summarizing quantification of CD133+ cells in 3 primary GBM cultures (mean of HuTuP13, 176, and 197) (right). (**C**,**D**) Representative contour plots displaying the combined expression of Nestin and Sox2 in HuTuP197 cells after 72 h of treatment with TSA (1 μM) and SAHA (2 μM) (**C**) and relative quantification of Nestin+ and Sox2+ cells in 3 different cell cultures (mean of HuTuP61, HuTuP176, and HuTuP197) (**D**). * *p* < 0.05, ** *p* < 0.01 by One-way ANOVA multiple comparison test. (**E**) Limiting dilution assay of GBM cells (HuTuP13) upon short-term exposure (24 h) to HDI (5 μM). Initiating cell frequency of cells in each condition is reported. #: number (**F**) Western blot analysis displaying the levels of TCF4 expression in pcDNA3.1 and pcDNA3.1-TCF4 (Myc-tagged) transiently transfected GBM cells (HuTuP197). The original Western blot images included in (**F**) are provided in Supplementary Figure S1D. (**G**) Bar graph summarizing quantification of CD133+ in GBM cells as in (**F**), treated with TSA (1 μM) and SAHA (2 μM) for 48 h. * *p* < 0.05, ** *p* < 0.01 by paired *t* test.

Previous results from our group demonstrated that TCF4 is a key transcriptional co-factor in modulating GBM stem cell differentiation status. Indeed, hMW TCF4 proteins act as inhibitors of Wnt signaling-induced gene transcription; thus, sustaining a CSCs phenotype [9]. Based on the knowledge that TSA and SAHA can induce a strong downregulation of TCF4 [15], we investigated if these compounds could also modulate TCF4 levels in GBM. To this end, we treated patient-derived GBM cells with TSA and SAHA for 24 h and confirmed that they were also able to strongly reduce TCF4 protein levels in our patient derived cells (Figure 1A and Supplementary Figure S1A,B). In parallel, HDI administration for 72 h was able to strongly decrease the expression of the neural stem cell markers CD133, Nestin, and Sox2 (Figure 1B–D). Indeed, as a functional validation of these data, a short-term exposure to HDI was sufficient to significantly decrease GBM stem cell frequency, as demonstrated by in vitro limiting dilution assays (Figure 1E and Supplementary Figure S1C).

These results agree with the anticipated hypothesis that HDI-mediated TCF4 inhibition could weaken the stem-like properties of GBM cells. In order to definitively support the already described pro-oncogenic function of TCF4 [9] and further sustain its involvement in the HDI-mediated reduction of the GBM stem cell phenotype, we treated GBM cells, transiently over-expressing a Myc-tagged TCF4, with both TSA and SAHA, as described, and demonstrated that increased levels of hMW TCF4 are sufficient to partially, although significantly, counteract the previously reported dramatic reduction of CD133 levels upon HDI exposure (Figure 1F,G and Supplementary Figure S1D,E).

### 3.2. HDI Treatment Impairs GBM Cell Proliferation and Synergize with TMZ

**Figure 2:**
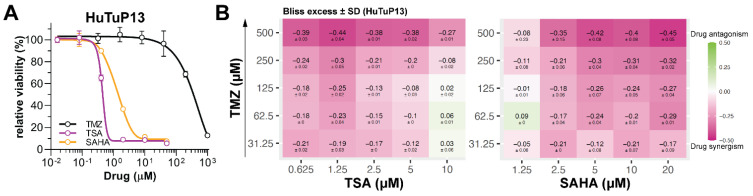
HDI affect GBM cell viability/proliferation and synergy with TMZ. (**A**) Dose-response viability curves of HuTuP13 GBM cells exposed to scalar doses of TSA, SAHA, or TMZ for 72 h. (**B**) Heatmaps displaying Bliss excess value matrixes of TSA (**left**) and SAHA (**right**) response in GBM cells (HuTuP13) when combined with TMZ. SD: standard deviation.

Based on the knowledge that GBM cells endowed with reduced stem-like properties, or even induced to differentiate, display increased sensitivity to standard chemotherapeutic agents [8,30], we investigated the effects of HDI treatment, in terms of cell viability/proliferation, in several primary GBM cultures (Supplementary Table S1) and compared their efficacy with the gold standard GBM therapeutic TMZ. TSA and SAHA demonstrated a higher efficacy relative to TMZ in all the primary GBM cells tested (Figure 2A and Supplementary Figure S2A). Indeed, both HDI displayed a significantly lower half maximal effective concentration (EC_50_) compared to TMZ, which proved to be almost ineffective. In particular, TSA demonstrated higher activity than SAHA, which, in general, achieved similar effects only at higher concentrations (Supplementary Table S2). Moreover, these experiments allowed finding HDI doses to be used for further experiments aimed at the molecular characterization of HDI-induced effects. Indeed, additional Annexin/PI staining of HDI treated GBM cells disclosed that neither 24 h of 1–5 μM TSA/SAHA, nor 1 μM TSA or 2 μM SAHA exposure for 72 h affected GBM cell viability (Supplementary Figure S2B,C). In order to assess the potential sensitizing effect of HDI administration when combined with TMZ, we treated GBM cells with TSA and SAHA, as single agents or in combination with TMZ using a 5 × 5 drug dilution matrix, according to the Bliss independence method [26]. Bliss excess values clearly demonstrated that both TSA and SAHA synergized with TMZ; thus, enhancing the response of GBM cells to the chemotherapeutic administration (Figure 2B).

### 3.3. TSA and SAHA Suppress HIF-1α and Wnt Signaling Activation

Our results indicate that both TSA and SAHA were able to reduce TCF4 levels in GBM cells, leading to an impairment of cell stemness. Moreover, it is now a defined paradigm that GBM differentiated cells are more sensitive to chemotherapeutics administration compared to their relative stem cell compartment [8,30]. In addition, we previously demonstrated that TCF4 may act as a transcriptional inhibitor of GBM cell differentiation, by counteracting the pro-neuronal stimuli exerted by a Wnt signaling-induced HIF-1α/β-catenin complex activation, through TCF1 [9]. 

**Figure 3:**
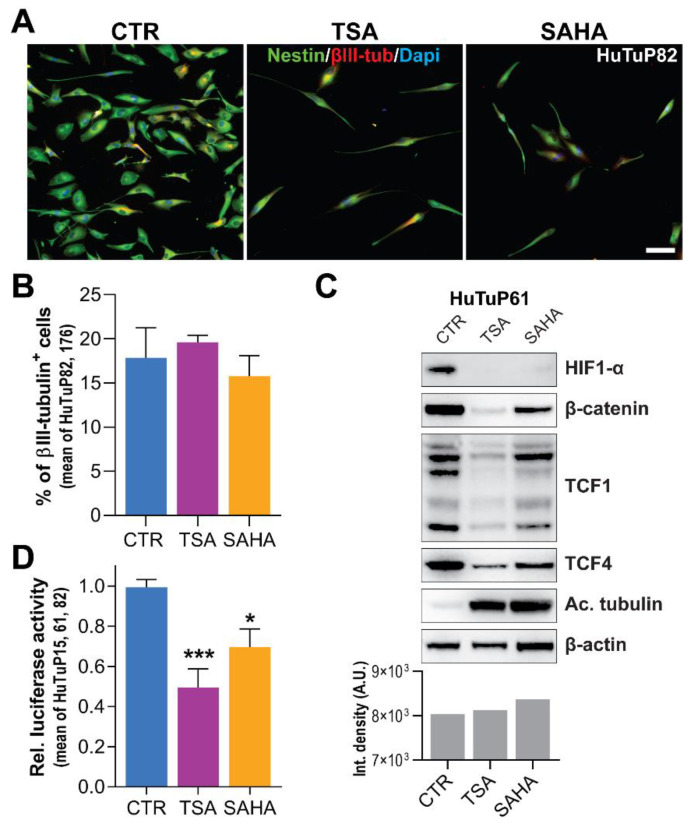
Reduction of GBM cell stemness is not accompanied by acquisition of a differentiated phenotype due to Wnt signaling suppression. (**A**) Representative immunofluorescence images displaying Nestin (green) and βIII-tubulin (red) expression of HuTuP82 cells after 5-d exposure to TSA (0.2 μM) and SAHA (0.5 μM). Cell nuclei were counterstained with Dapi (blue). Original magnification 10×; bar: 30 μm. (**B**) Quantification of βIII-tubulin+ cells by immunofluorescence as in (**A**) (mean of HuTuP82 and HuTuP176 GBM cells). (**C**) Western blot analysis of indicated proteins extracted from HuTuP61 cells treated for 24 h with TSA (5 μM) and SAHA (5 μM) (upper panel). The original Western blot images included in (**C**) are provided in Supplementary Figure S3B. Bar graph reporting total protein quantitation (integrated density) by Coomassie staining of WB samples (bottom panel). (**D**) Bar graph reporting relative luciferase activity of GBM cells (mean of HuTuP15, 61 and 82) transfected with BAT-lux plasmid and then treated for 24 h with HDI (5 μM). A.U.: arbitrary units. * *p* < 0.05, *** *p* < 0.001 by One-way ANOVA multiple comparison test.

Based on this premise, we hypothesized that HDI treatment of GBM cells, besides inhibiting stemness, would engage a neuronal differentiation process. However, immunofluorescence analysis, despite confirming a strong reduction of the neural stem cell marker Nestin, did not evidence the acquisition of neuronal traits, either at 72 h or at 5 days (d) post-treatment, suggesting that TSA and SAHA (through the inhibition of TCF4 levels) do not exert neuronal pro-differentiating effects, per se (Figure 3A,B and Supplementary Figure S3A). According to this result, a deepened analysis of the molecular players, already described as participating in regulating the neuronal differentiation in GBM cells [9], revealed that a short-term HDI administration is sufficient to potently reduce, besides TCF4, the protein levels of HIF-1α, β-catenin, and TCF1 (Figure 3C and Supplementary Figure S3B–D). These data are in accordance with the observed lack of HDI-induced neuronal differentiation, which is, thus, consistent with the absence of a functional HIF-1α/β-catenin/TCF1 transcriptional complex. As a functional validation of these results, HDI treatment of Wnt reporter-transfected cells induced a significant inhibition of Wnt signaling-dependent transcriptional activity (Figure 3D). 

### 3.4. Gene Expression Profiling Reveals an HDI-Induced Modulation of Cell Cycle and Cell Migration-Associated Transcriptional Programs

**Figure 4:**
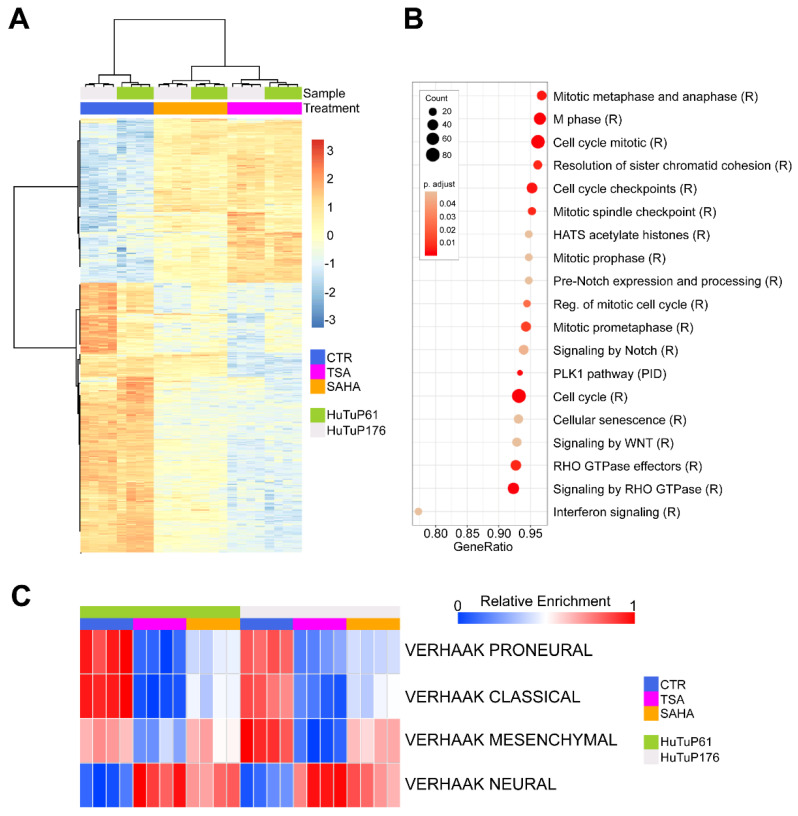
TSA and SAHA significantly modulate GBM cell transcription. (**A**) Heatmap displaying hierarchical clustering of control and HDI-treated GBM cells (5 μM for 24 h) according to the top variable transcripts. (**B**) Dot plot showing significant (FDR q value < 0.05) transcriptional enrichments (C2cp MSigDB) of common DEGs between TSA and SAHA treated cells as in (**A**). (**C**) Heatmap displaying relative enrichment of each sample subjected to GEP in the four different GBM molecular subtypes [2] from the C2cgp MSig database.

The data obtained so far highlight the marked activity of TSA and SAHA in turning off Wnt signaling, with a consequent impairment of GBM cell stemness and viability, without stimulating differentiation toward a neuronal cell fate. To further characterize the effects induced by HDI treatment in GBM cells, we profiled whole cell transcriptome in two HDI-treated primary GBM cultures. TSA or SAHA-treated cells displayed a clear-cut difference in terms of transcriptional features relative to matched control cells (Figure 4A). In particular, we identified 1751 differentially expressed genes (DEGs) between control and TSA-treated cells, and 1730 DEGs between control and SAHA-treated cells. Importantly, 1233 DEGs (71.27% of TSA-modulated genes and 70.42% of SAHA-modulated genes) resulted as commonly affected by TSA and SAHA treatments (Supplementary Figure S4A and Supplementary Table S3). 

Commonly perturbated DEGs were then subjected to a pathway enrichment analysis, showing down-regulated transcripts as significantly enriched for three main cellular processes, including (i) cell cycle components and dynamics; (ii) Rho GTPase activity; and (iii) interferon signaling pathway (Figure 4B). In addition, the analysis of further potential enrichments in gene sets from the C2cgp database confirmed a significant modulation of peculiar transcriptional features associated with the HDI response, a consistent inhibition of cell cycle/mitotic genes, and a reduction of interferon signaling targets (Supplementary Figure S4B). Intriguingly, single sample GSEA (ssGSEA) underlined a shared HDI-induced transcriptional shift from a mixed transcriptional subtype, toward a Neural, molecular subgroup (Figure 4C). Of note, despite the SAHA treatment resulting as less effective in suppressing peculiar Mesenchymal transcriptional features, the general HDI-dependent acquisition of Neural subtype traits is highly relevant, since Neural GBM tumors are generally considered to bear less aggressive characteristics and have a longer life expectancy [2,31].

### 3.5. HDI Inhibit GBM Cell Proliferation by Acting at the G_2_/M Cell Cycle Checkpoint

**Figure 5:**
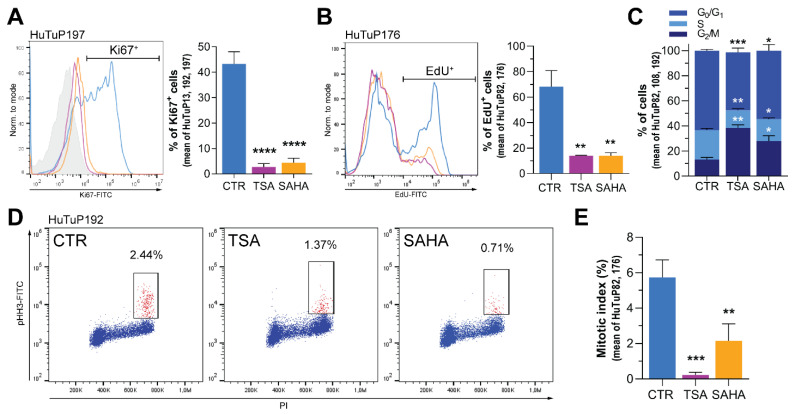
HDI inhibit proliferation by preventing GBM cells from proceeding in the cell cycle. (**A**) Representative flow cytometric evaluation of Ki67 in control and TSA (1 μM)/SAHA (2 μM)-treated GBM cells (72 h; HuTuP197) (left) and graph summarizing its quantification in 3 primary GBM cultures (mean of HuTuP13, 192, and 197) (right). (**B**) Representative flow cytometric evaluation of EdU incorporation in control and HDI treated GBM cells (72 h; HuTuP176) as in (**A**) (left), and graph summarizing its quantification in 2 primary GBM cultures (mean of HuTuP82 and 176) (right). (**C**) Bar graph displaying the relative distribution of GBM cells (mean of HuTuP82, 108 and 192) in the G0/G1, S, and G2/M phases of the cell cycle. (**D**) Representative PI/pHH3 cytofluorimetric staining in GBM cells (HuTuP192). (**E**) Bar graph displaying mitotic index values in control and HDI-treated GBM cells (mean of HuTuP82 and 176). * *p* < 0.05, ** *p* < 0.01, *** *p* < 0.001, **** *p* < 0.0001 by one-way ANOVA multiple comparison test.

In order to functionally validate transcriptional data and better characterize the effects of TSA and SAHA on GBM cell proliferation, we analyzed the expression of the proliferation marker Ki67 upon 72 h HDI exposure, demonstrating its strong and significant reduction by both flow cytometry and immunofluorescence (Figure 5A and Supplementary Figure S5A). In order to functionally validate this result, we performed additional EdU incorporation assays, which clearly demonstrated an HDI-induced impaired proliferative capacity of GBM cells (Figure 5B). Analysis of cell cycle phases further deepened these effects, showing that both TSA and SAHA were able to induce a significant accumulation of cells in the G2/M phase of the cell cycle, along with a concomitant reduction of the S phase (Figure 5C). Moreover, despite significantly increasing the proportion of cells in the G_2_/M phase, HDI treatment slightly but consistently decreased the amount of phospho-Histone H3 (p-S10-HH3)^+^ cells, suggesting their action at the level of the G2-M checkpoint; thus, preventing cells from entering mitosis (Figure 5D and Supplementary Figure S5B). Accordingly, HDI-treated cells displayed a significant reduction of the number of cells undergoing mitosis, as shown by the analysis of mitotic index (Figure 5E), further supporting this hypothesis. Altogether, these data confirm the gene expression profiling results, demonstrating that TSA and SAHA strongly inhibit cell proliferation of GBM cells by counteracting their ability to proceed within the cell cycle.

### 3.6. TSA and SAHA Inhibit GBM Cell Motility by Affecting RhoA and Interferon-Dependent Pathways

**Figure 6:**
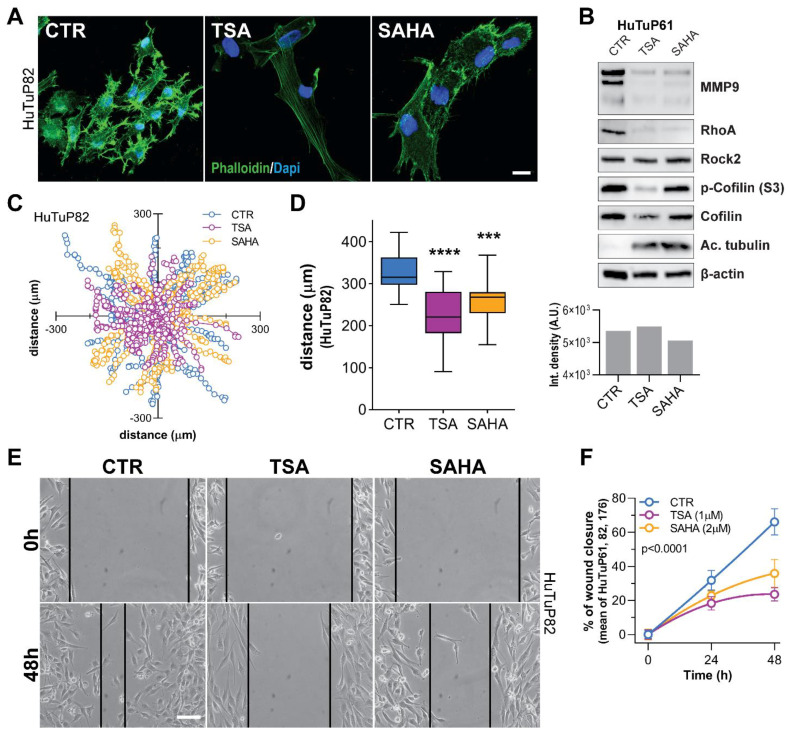
HDI induce cytoskeletal rearrangements and RhoA-GTPase-dependent inhibition of cell motility/migration. (**A**) Representative immunofluorescence images of HuTuP82 GBM cells displaying cytoskeletal (phalloidin, green) alterations associated with TSA (1 μM) and SAHA (2 μM) treatments for 72 h. Cell nuclei were counterstained with Dapi (blue). Original magnification 20×; bar: 10 μM. (**B**) Western blot analysis of proteins (as indicated) extracted from HuTuP61 cells treated for 24 h with TSA (5 μM) and SAHA (5 μM) (upper panel), and bar graph reporting total protein quantitation (integrated density) by Coomassie staining of same samples). The original Western blot images included in (**B**) are provided in Supplementary Figure S6B. (**C**,**D**) Graph representing the normalized (x, y) trajectories of control and HDI-treated (1 μM TSA and 2 μM SAHA) GBM cells moving within the plate in a 16-h timespan (**C**), and box plot summarizing the total length covered by cells in the same time interval (HuTuP82) (**D**). *** *p* < 0.001, **** *p* < 0.0001 by one-way ANOVA multiple comparison test. (**E**,**F**) Representative images demonstrating the inhibitory effect displayed by HDI (1 μM TSA and 2 μM SAHA) on the ability of GBM cells (HuTuP82) to close the wound during a scratch assay (**E**), and relative quantification of the scratch closure assay performed in the HuTuP61, HuTuP82, and HuTuP176 GBM cells (**F**). Original magnification 10×; bar: 50 μM.

One of the factors making GBM such a complex tumor to treat, is its elevated ability to migrate and infiltrate the surrounding tissues. Indeed, tumor cells can be found at a distance from the site of tumor onset, even at diagnosis [32]. Gene expression profiling (GEP) data disclosed a significant downregulation of transcripts related to the Rho-GTPase family of proteins, which are known to regulate cell adhesion dynamics [33], cytoskeletal processes [34], cell migration and invasion [35], and even cell cycle progression [34,36]. Indeed, by evaluating cell morphology and the organization of actin fibers through Phalloidin staining, we observed a strong reduction of lamellipodia and filopodia and a strong reorganization of actin filaments in HDI-treated cells (Figure 6A and Supplementary Figure S6A). Starting from this evidence, we analyzed the levels of RhoA and its downstream effectors involved in regulating cell motility and matrix metallopeptidase (MMP) activity. Western blot confirmed that both TSA and SAHA possess the ability to reduce RhoA levels and weaken the activation (phosphorylation) of its intracellular effector Cofilin (Figure 6B and Supplementary Figure S6B,C). In addition, also MMP9 resulted as strongly downregulated by HDI exposure (Figure 6B and Supplementary Figure S6B,C).

Given the observed action of TSA and SAHA in reducing RhoA-dependent signaling, we then performed a migration assay to functionally validate their anticipated role against cell motility. As a result, time-lapse imaging of HDI-treated cells disclosed a significant reduction in the complexity of cell trajectory during movements and in the total distance covered by cells inside the wells (Figure 6C,D and Supplementary Figure S6D,E). As a further functional confirmation of this result, HDI significantly reduced the ability of GBM cells to move into and close the wounded monolayer during scratch assays performed in multiple primary cultures (Figure 6E,F).

**Figure 7:**
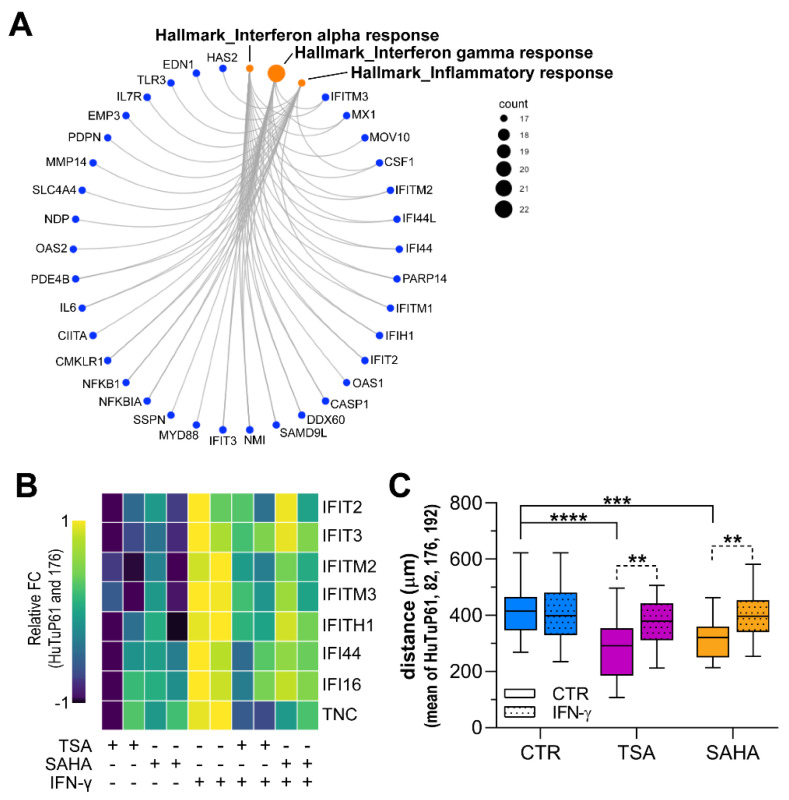
HDI-induced inhibition of cell motility is partially dependent on IFN target gene suppression. (**A**) Circle plot representing HDI-induced DEGs contributing to a significant negative enrichment (FDR q value < 0.05) of the transcriptional Hallmarks (Hallmarks gene sets from MSigDB) correlated to the IFN-α/γ and inflammatory responses. (**B**) Heatmap summarizing the relative expression of a series of IFN/inflammation signaling target genes as indicated when GBM cells (HuTuP61, 176) were exposed to TSA (5 μM), SAHA (5 μM), IFN-γ (1 μg/mL), or a combination of them for 24 h. (**C**) Box plot summarizing the total length covered by cells (HuTuP61, 82, 176, 192, 197) when treated with TSA (1 μM) and SAHA (2 μM) (combined or not with 1 μg/mL IFN-γ) during a 16-h live imaging experiment. ** *p* < 0.01, *** *p* < 0.001, **** *p* < 0.0001 by *t* test or one-way ANOVA multiple comparison test.

Enrichment analyses of GEP-identified DEGs revealed the inhibition of the interferon (IFN)-regulated pathways in response to HDI treatment (Figure 4B and Supplementary Figure S4B). Moreover, an additional over-representation test performed in the Hallmarks database, intriguingly, indicated that both inflammatory and IFN-α/γ response genes were subjected to a significant HDI-dependent attenuation (Figure 7A). Since previous studies demonstrated that inflammation and IFN signaling, besides regulating leukocyte recruitment and activation, play a pivotal role in sustaining the pro-migrating/invasive capability of cancer cells, including glioma [16], we hypothesized that this inhibition of IFN/inflammatory genes could contribute to the impairment of GBM cell motility exerted by TSA and SAHA. In order to validate this intriguing indication, we first demonstrated that a short-term TSA/SAHA treatment was sufficient to significantly downregulate both the basal and the IFN-γ-induced expression of a selection of IFN responsive genes, including IFIH1, IFIT2, IFIT3, IFITM2, IFITM3, IFI16, IFI44, and TNC (Figure 7B and Supplementary Figure S7A). Based on these promising results, we functionally assayed, through live imaging microscopy, the involvement of IFN-γ in sustaining the enhanced migratory potential displayed by GBM cells. Of note, IFN-γ stimulation, besides not impacting on the still prominent migrating potential of GBM cells, was able to rescue the already reported HDI-dependent mitigation of cell movement complexity (not shown) and the total distance covered by cells (Figure 7C), without affecting GBM cell viability or their response to TSA and SAHA (Supplementary Figure S7B).

In conclusion, all these data support the hypothesis that HDI can weaken GBM cell migratory potential by acting at multiple levels on cell transcription, including a modulation of the RhoA-GTPase signaling and multiple IFN pathway target genes. 

## 4. Discussion

GBM is currently the most frequent malignant neoplasm of the central nervous system [37]. Since the introduction of the Stupp protocol, which comprises the concomitant use of radiotherapy and the administration of the alkylating prodrug TMZ after surgery [38], not much progress has been made in improving the survival rates of patients. Based on this, in the recent years, researchers have attempted to better characterize the molecular basis of the intrinsic GBM aggressiveness, providing a better comprehension of the role played by (i) genetic/epigenetic alterations [1,39,40], (ii) restricted subpopulations of tumor propagating cells and CSCs [5,7], and (iii) GBM intra/intertumoral heterogeneity [8,41], in sustaining tumor growth and therapeutic resistance. In this context, a seminal study from Verhaak et al. disclosed that GBM tumors can be classified into four distinct transcriptional subtypes, namely the Neural, Proneural, Classical, and Mesenchymal molecular subgroups; bearing peculiar genetic and transcriptional aberrations that finally reflect on tumor phenotype and response to treatment schedules [2]. Although later studies provided additional clues for better interpreting the relationship between transcriptional signatures and patient outcome [42], it is widely recognized that Mesenchymal GBM patients display poor prognoses and response to therapy. In this context, we show that HDI treatment induces a significant transcriptional shift in GBM cells, promoting the transition from a mixed Proneural/Classical/Mesenchymal molecular subtype, to the Neural class, characterized by increased phenotypic differentiation and transcriptional similarities with normal brain tissues [2], with potential consequences for tumor cell behavior and CSC propagating capacity.

After their identification in different brain tumors, including GBM [43,44], CSC were then demonstrated to be resistant to chemotherapy [5] and radiotherapy [45], highlighting their leading role in sustaining disease progression and recurrence in GBM. In this context, we previously demonstrated that the GBM hypoxic core is enriched in chemotherapy-resistant CSC [8], which, once pushed to differentiation, becomes much more sensitive to standard drug treatments [10,30]. This assumption is also relevant for interpreting the data provided by this study. Indeed, we recently described an intriguing mechanism by which the interplay between the HIF-1α and the Wnt pathways may operate as an upstream regulator of the balance between stemness (through TCF4) and neuronal differentiation (through HIF-1α/TCF1) in GBM [9]. Based on this knowledge and a recent study describing that TSA and SAHA were able to reduce TCF4 levels by acting on its transcription and protein degradation in colorectal cancer cells [15], we hypothesized that a HDI-induced suppression of TCF4 levels cells could ignite neuronal differentiation in GBM and sensitize them to chemotherapeutics. Here, it seems quite clear that, even if they are not able to promote the anticipated neuronal differentiation of GBM cells, due to a non-selective suppression of several Wnt signaling components, HDI significantly interfere with their phenotypic and functional stem cell features. In this context, and from a molecular point of view, we confirmed that TCF4 is clearly involved in sustaining GBM cell stemness, and its dramatic reduction induced by HDI could represent, if not the unique, at least one relevant mechanism by which TSA and SAHA are able to affect GBM stemness. Of note, this effect may also provide a reasonable explanation of the strong synergism we observed between HDI and TMZ, suggesting an additional benefit of their use in the treatment of GBM.

Despite the data obtained within this study demonstrating that HDI, not only affect TCF4 levels, but also suppress Wnt signaling activation; thus, making them not suitable for achieving a specific TCF4 inhibition, their promising effects displayed during GBM cell treatment encouraged us to better understand the mechanisms by which TSA and SAHA exert their anti-cancer action in GBM. As anticipated by the GEP results, HDI treatment induced a significant increase of the proportion of GBM cells in the G_2_/M phase. In this context, a concomitant slight reduction of pHH3 levels evidenced that HDI may potentially act at the G_2_/M checkpoint. Although HDI have already been shown to interfere with chromosome passenger complex-induced HH3 phosphorylation; thus, reducing its levels in functional mitotic cells [46], a further analysis of the mitotic index confirmed a significant reduction of cells undergoing mitosis, corroborating the hypothesis of the G_2_/M checkpoint involvement. Indeed, inhibition of cell cycle dynamics by HDI, by acting on the G_1_/S or the G_2_/M transition checkpoints, depending on the specific drug used and the cellular context, has been extensively reported in other tumors, appearing to be one of the mechanisms sustaining their efficacy [47,48,49,50].

A major hallmark of GBM is its great ability to disseminate and invade the surrounding normal brain tissue; thus, preventing a complete and therapeutically efficient surgical resection of the mass, and, finally, negatively impacting on the risk of relapse and patient cure. Despite the therapeutic advances provided by the intra-operative detection of disseminated cells through 5-aminolevulinc acid guidance [6,51], in recent years, the study of the GBM cell invasive potential has become of primary importance, to identify new pharmacological targets to improve patient survival. GBM migration and invasion processes, even if still under investigation, are generally linked to three main signaling networks involving Hephrin receptors, Rho-GTPases, and Casein kinase 2 (CK2) [34,35,36,52]. In this context, our results demonstrating that HDI possess the ability to reduce the activation of the RhoA–GTPase axis is unquestionably relevant, particularly with reference to the RhoA intracellular effector, Cofilin. Indeed, this protein plays a pivotal role in modulating the migratory capabilities of cells, by controlling the dynamics of actin filament polymerization and rearrangement [53], which support several cellular motility structures. Accordingly, HDI-induced rearrangement of the actin cytoskeletal framework was strictly associated with the loss of focal adhesions and filopodia in GBM cells, with reported functional consequences on cell motility. In addition, the observed reduction of MMP9 levels could potentially contribute to a combined suppression of both cell motility and tissue invasion.

As consistently indicated by multiple enrichment analyses, the HDI-induced DEGs list includes a series of IFN-α/γ and inflammatory response target genes that are significantly downregulated by both TSA and SAHA. Intriguingly, some of these genes, particularly the interferon-induced proteins with tetratricopeptide repeats (IFIT) and the interferon-induced transmembrane proteins (IFITM), have already been reported to be directly correlated to multiple processes, such as cell survival and proliferation, migration, and invasion in several cancers, including gliomas [16,17,18,24,54,55,56]. In particular, several IFN-induced IFIT proteins have been directly correlated to a disseminating and pro-metastatic phenotype in lung adenocarcinoma, squamous cell carcinoma, bladder, and prostate cancers [20,54,55,57]. In addition, these IFN-induced factors are suggested to participate at multiple levels in the transduction/modulation of various signals, including those coming from EGFR [16] or converging in the JNK–STATs axis [56]. Even if a potent IFN-γ-induced over-expression of a series of these genes had no impact on the response of GBM cells to HDI; nevertheless, they retained a significant action on cell motility, by counteracting the HDI-dependent shrinkage of cell movements already observed in this study. These latter results pinpoint a specific role played by the inflammatory response genes in GBM that, even if they are only partially inhibited by HDI upon IFN stimulation, may harbor a threshold-dependent regulatory switch in the control of GBM cell motility/invasion, which needs further investigation. 

## 5. Conclusions

The results obtained within this study demonstrate that HDI TSA and SAHA, despite not acting as specific TCF4 inhibitors as initially hypothesized, actually possess a consistent anti-cancer efficacy in GBM cells, demonstrated by (i) a strong sensitizing effect on TMZ response; (ii) a potent impairment of CSC phenotype and self-renewal; (iii) an anticipated interference of cell proliferation and cell cycle progression; and (iv) a therapeutically relevant inhibition of cell motility/migration through a combined impairment of the Rho–GTPase axis-dependent action on cytoskeletal dynamics and the migratory potential sustained by the expression of IFN response genes. Since Vorinostat (SAHA) has already received approval for the treatment of various cancers, our data suggest that would probably be worthwhile to better define the translational potential of its use in GBM patients; however, not before a more detailed investigation of its in vivo effectiveness in appropriate models and the identification of potential undesirable effects.

## Data Availability

Gene expression data generated within this study have been deposited in the GEO database under Series Accession Number GSE191126 and are accessible without restrictions.

## Supplementary Materials

The following supporting information can be downloaded at: https://www.mdpi.com/article/10.3390/cancers14081897/s1, Supplementary Table S1: General characteristics of patients from which GBM primary cultures used within this study were derived. Supplementary Table S2: EC_50_ values of TSA and SAHA in all GBM cultures tested. Supplementary Table S3: List of genes commonly perturbated by TSA and SAHA treatment. Supplementary Table S4: List of primer sequences used within the study. Supplementary Figure S1: TSA/SAHA treatment reduces TCF4 levels and impairs the functional stem cell properties of GBM cells. Supplementary Figure S2: HDI affect GBM cell viability/proliferation. Supplementary Figure S3: HDI impair Wnt signaling activation in GBM cells. Supplementary Figure S4: TSA and SAHA commonly perturbated genes display peculiar transcriptional enrichments in the C2cgp database. Supplementary Figure S5: HDI reduce GBM cell proliferation. Supplementary Figure S6: HDI reduce GBM cell motility by acting on the RhoA-GTPase signaling. Supplementary Figure S7: HDI inhibit IFN pathway target genes involved in cell motility and invasion.
