# Supplementary material for: Histone Deacetylase Inhibitors Impair Glioblastoma Cell Motility and Proliferation
Source: Cancers (Basel). 2022 Apr 9;14(8):1897. doi: 10.3390/cancers14081897 (PMC9027190; doi:10.3390/cancers14081897)
Supplement: Supplementary file 1 [file cancers-14-01897-s001.zip › cancers-1554593-supplementary-done.pdf]

cancers

MDPI

Supplementary Materials

Table S1. General characteristics of patients from which GBM primary cultures used within this study have been derived.

Patient ID	Diagnosis	WHO grade	Gender	Age (y)
**HuTuP13**	GBM	IV	Male	67
**HuTuP15**	GBM	IV	Female	76
**HuTu47**	GBM	IV	Female	81
**HuTuP61**	GBM	IV	Female	70
**HuTuP82**	GBM	IV	Male	50
**HuTuP108**	GBM	IV	Male	62
**HuTuP176**	GBM	IV	Male	59
**HuTuP192**	GBM	IV	Male	62
**HuTuP197**	GBM	IV	Male	48

Table S2. EC_{50} values calculated for TSA, SAHA and TMZ in the indicated primary GBM cell cultures.

Patient ID	EC_{50} TMZ (µM)	EC_{50} TSA (µM)	EC_{50} SAHA (µM)
**HuTuP13**	358.37	0.43	1.42
**HuTuP15**	790.79	1.60	2.16
**HuTuP61**	>1000	2.51	11.01
**HuTuP108**	>1000	1.28	3.04
**HuTuP176**	>1000	1.55	3.85
**HuTuP192**	155.15	1.77	1.22
**HuTuP197**	>1000	0.94	1.81

Table S3. List of genes commonly perturbated by TSA and SAHA treatment (provided as a separate excel file).

Table S4. List of primer sequences used within the study.

Gene	Sequence (5′-3′)	Amplicon (bp)
IFITM2 forward	TGGGCTTCATAGCATTCGCGT	197
IFITM2 reverse	ATGCCTCCTGATCTATCGCTGGG	
IFITM3 forward	CCATGTCGTCTGGTCCCTGTT	234
IFITM3 reverse	TATCCATAGGCCTGGAAGATCAGCA	
IFIT2 forward	CCCTGCCGAACAGCTGAGAAT	240
IFIT2 reverse	TGCCTCGTTTTGCCCTTTGAG	
IFIT3 forward	CCCCTTCAGGCATAGGCAGT	173
IFIT3 reverse	CAACCACCACTGCAGGCTTC	
IFI16 forward	ATGGATGTAGTGGGGACAGGA	199
IFI16 reverse	GCTGTTCCTGGGGTAGCTTCA	
IFI44 forward	GAGAGATGTGAGCCTGTGAGGT	164
IFI44 reverse	GCAGCCCATAGCATTCGTCTC	
IFIH1 forward	CACGAAGCAAGCCAAAGCTGA	132
IFIH1 reverse	GCAAACTTCTTGCATGGCTCCT	
TNC forward	TCTGATGGGGGTGGATGGAT	134
TNC reverse	TTCAGGTTGTCCAGCCCAAG	
GUSB forward GUSB	GAAAATACGTGGTTGGAGAGCTCATT	101
reverse	CCGAGTGAAGATCCCCTTTTTA	

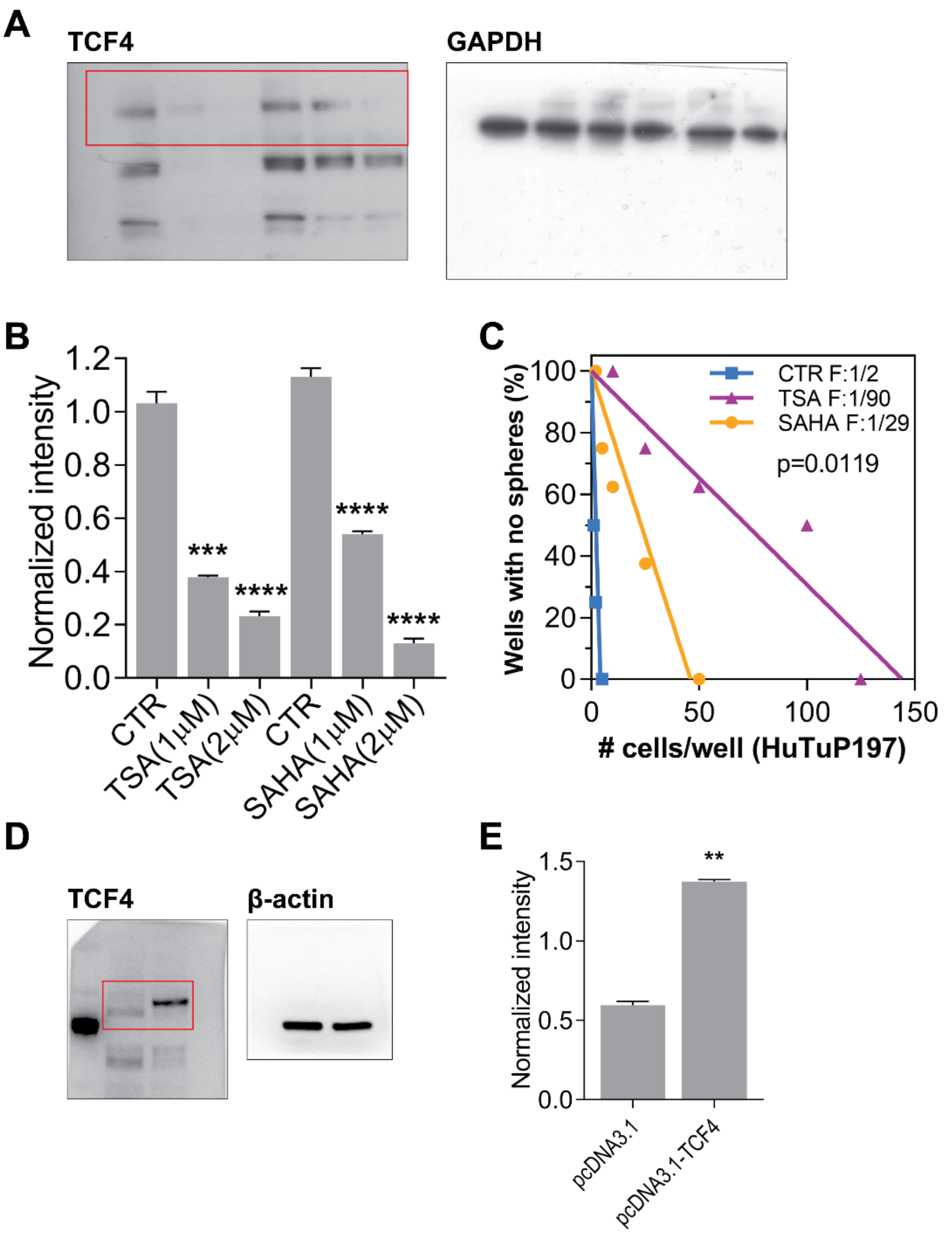

Figure S1. TSA/SAHA treatment reduces TCF4 levels and impairs functional stem cell properties of GBM cells. (**A**) Original Western Blot images included in Fig. 1A. Cropped WB bands displayed in Fig. 1A are highlighted by a red box. (**B**) Bar graph summarizing normalized intensity of western blot band densitometry from at least 3 independent experiments in which GBM cells have been treated with HDI (1–2 µM for 24 h). *** $p < 0.001$, **** $p < 0.0001$ by One-way ANOVA multiple comparison test. (**C**) Limiting dilution assay of GBM cells (HuTuP197) upon short-term exposure (24 h) to HDI (5 µM). Initiating cell frequency of cells in each condition is reported. (**D**) Original Western Blot images included in Fig. 1F. Cropped WB bands displayed in Fig. 1F are highlighted by a red box. (**E**) Bar graph summarizing normalized intensity of western blot band densitometry from at least 3 independent experiments in which GBM cells have been transiently transfected with pcDNA3.1 empty vector or pcDNA3.1-TCF4 and then used for CD133 flow cytometric evaluation. ** $p < 0.01$ by t test.

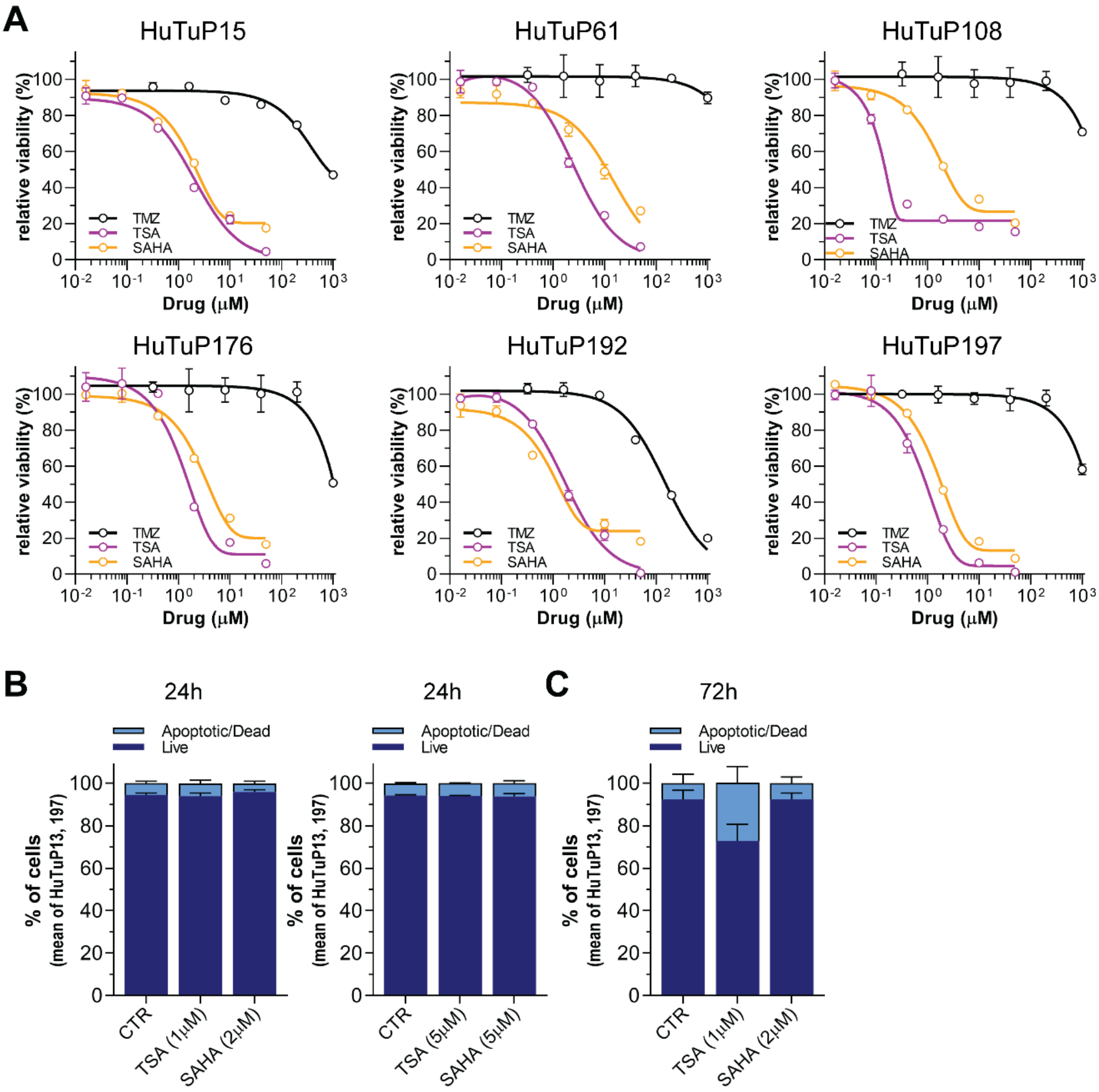

Figure S2. HDI affect GBM cell viability/proliferation. (**A**) Dose-response viability curves of 6 different GBM cultures exposed to scalar doses of TSA, SAHA or TMZ for 72hrs. (**B**,**C**) Bar graphs displaying Annexin/PI staining of 1–5 µM TSA/SAHA treated (**B**) or 1µM TSA/2µM SAHA treated (**C**) GBM cells for 24hrs and 72hrs, respectively (HuTuP13 and 197).

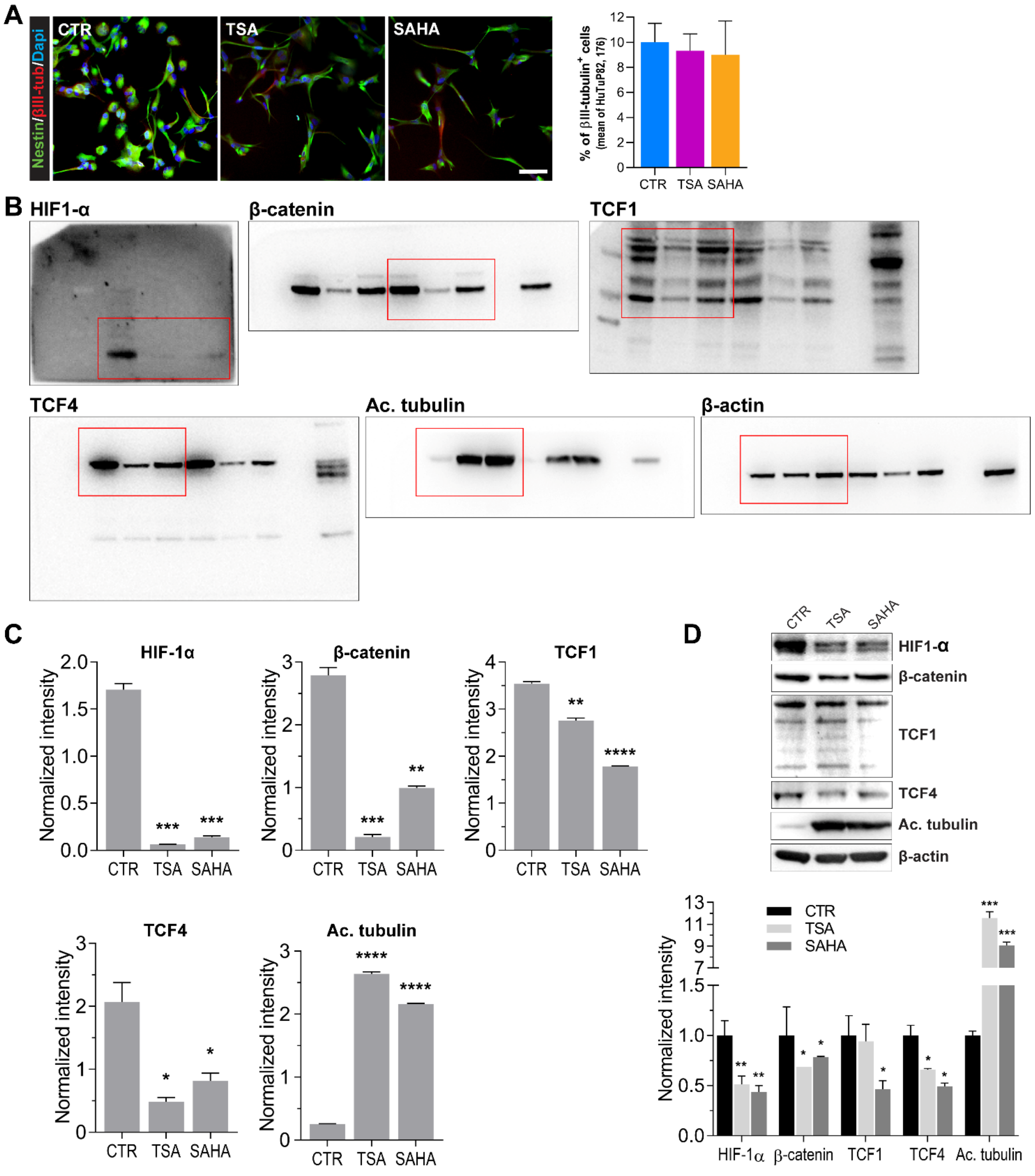

Figure S3. HDI impair Wnt signaling activation in GBM cells. (**A**) Representative immunofluorescence images displaying Nestin (green) and βIII-tubulin (red) expression of HuTuP176 cells after 72 h exposure to TSA (1 μM) and SAHA (2 μM) for 72 h. Cell nuclei have been counterstained with Dapi (blue). Original magnification 10×; bar: 20μm (left panels). Right panels show relative quantification of βIII-tubulin+ cells (mean of HuTuP82 and P176 cells). (**B**) Original Western Blot images included in Fig. 3C. Cropped WB bands displayed in Fig. 3C are highlighted by a red box. (**C**) Bar graphs summarizing normalized intensity of Western Blot band densitometry from at least 3 independent experiments as in (**B**), TSA (5 μM) and SAHA (5 μM) for 24 h. (**D**) Western Blot analysis of indicated proteins extracted from HuTuP13 GBM cells treated as in (**B**,**C**) and relative band densitometry from at least 3 independent experiments. * $p < 0.05$, ** $p < 0.01$, *** $p < 0.001$, **** $p < 0.0001$ by One-way ANOVA multiple comparison test..

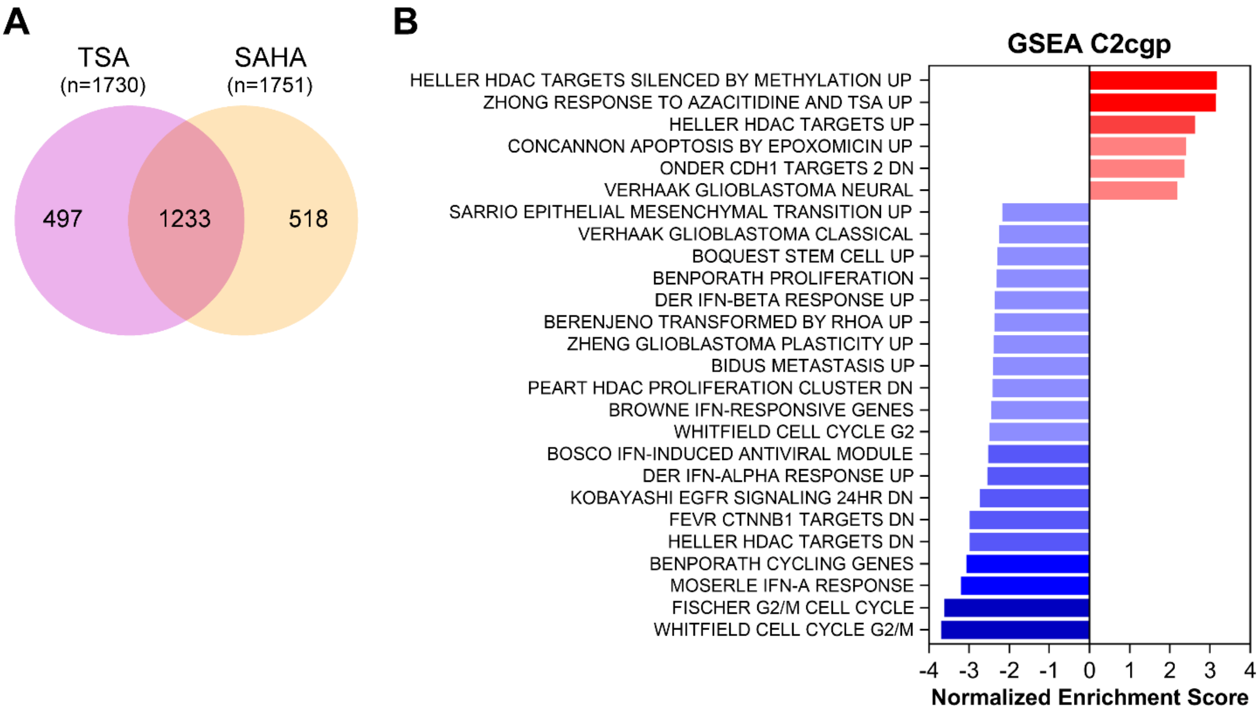

Figure S4. TSA and SAHA commonly perturbated genes display peculiar transcriptional enrichments in the C2cgp database. (**A**) Venn diagram identifying commonly DEGs between TSA and SAHA treatments. (**B**) Bar plot summarizing the significant (FDR q value < 0.05) transcriptional enrichments in gene sets from the C2cgp MSig database.

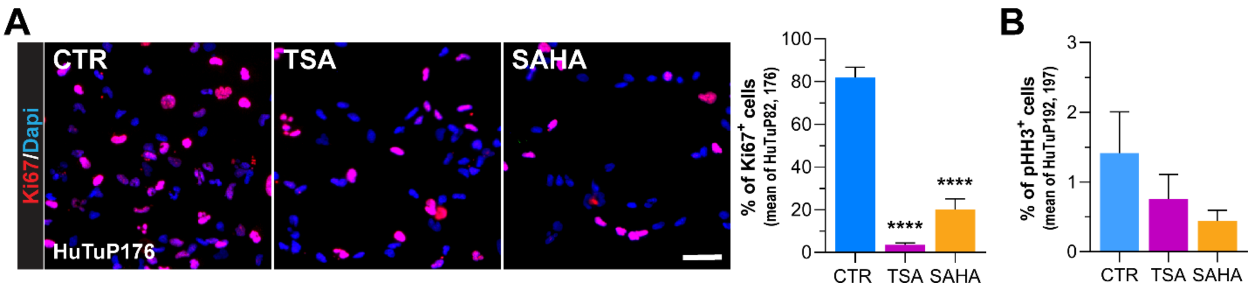

Figure S5. HDI reduce GBM cell proliferation. (**A**) Representative immunofluorescence images showing Ki67^{+} cells (red) in control and TSA (1 µM)/SAHA (2 µM) treated GBM cells (72 h; HuTuP176) (left panels). Cell nuclei have been counterstained with Dapi (blue). Original magnification 10×; bar: 20 µm. Bar graph showing the quantification of Ki67^{+} GBM cells (HuTuP82 and 176 by immunofluorescence) (right panel). (**B**) Bar graph showing the percentage of GBM cells (HuTuP192 and 197) demonstrating positivity for the M phase marker pHH3^{+} by flow cytometry. **** $p < 0.0001$ by One-way ANOVA multiple comparison test.

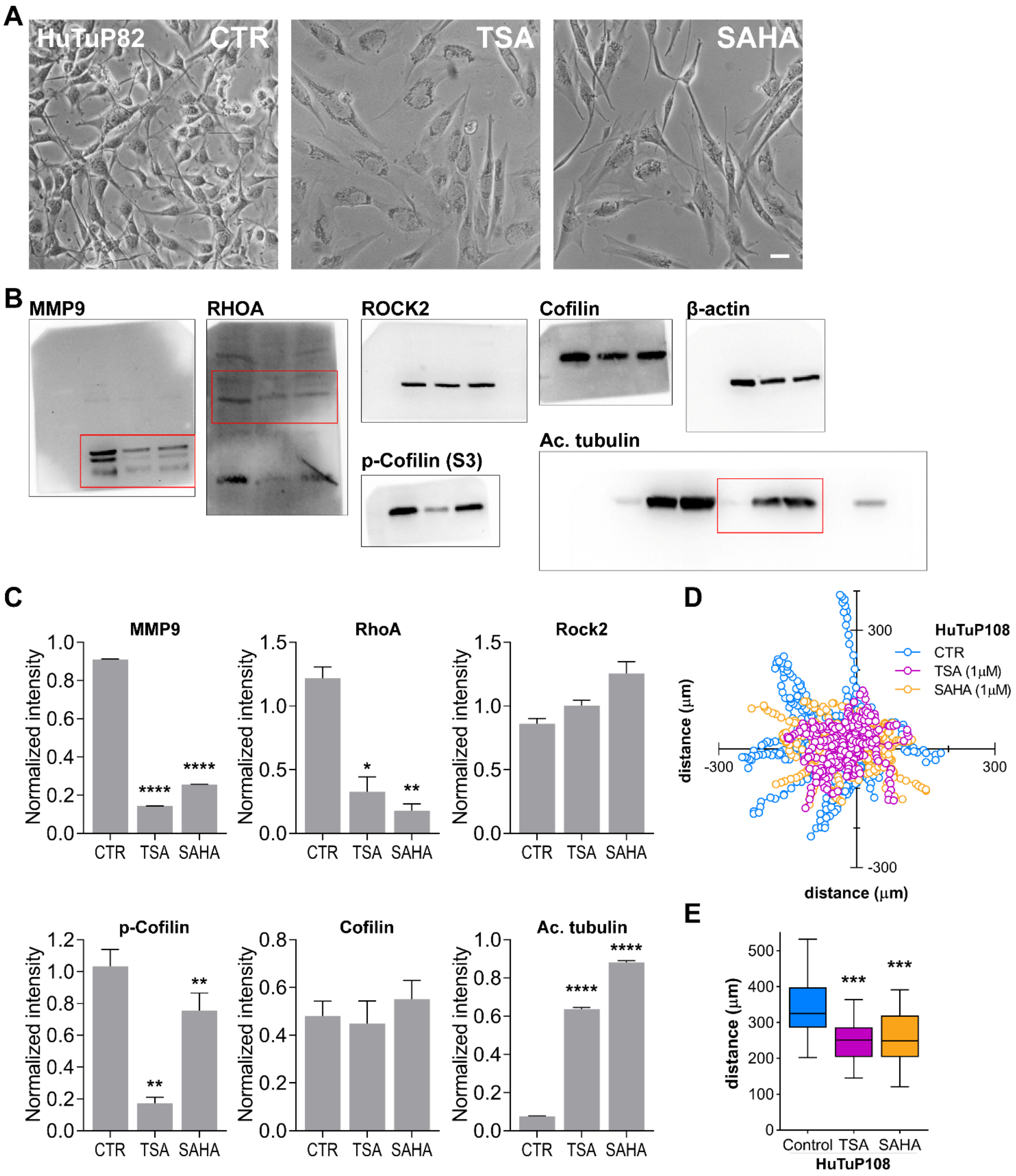

Figure S6. HDI reduce GBM cell motility by acting on the RhoA-GTPase signaling. (**A**) Representative brightfield images of morphological alterations occurring in HuTuP82 cells upon TSA (1 μM) or SAHA (2μM) treatment for 72 h. Original magnification 10×; bar: 10 μm. (**B**) Original Western Blot images included in Figure 6B. Cropped WB bands displayed in Figure 6B are highlighted by a red box. (**C**) Bar graphs summarizing normalized intensity of Western Blot band densitometry from at least 3 independent experiments in which GBM cells have been treated with TSA (5 μM) and SAHA (5 μM) for 24 h. (**D**,**E**) Graph representing the normalized x,y trajectories of control and HDI-treated GBM cells (1 μM TSA and 2 μM SAHA) moving within the plate in a 16hrs timespan (**D**) and box plot summarizing the total length covered by cells (**E**) (HuTuP108). * $p < 0.05$, ** $p < 0.01$, *** $p < 0.001$, **** $p < 0.0001$ by One-way ANOVA multiple comparison test.

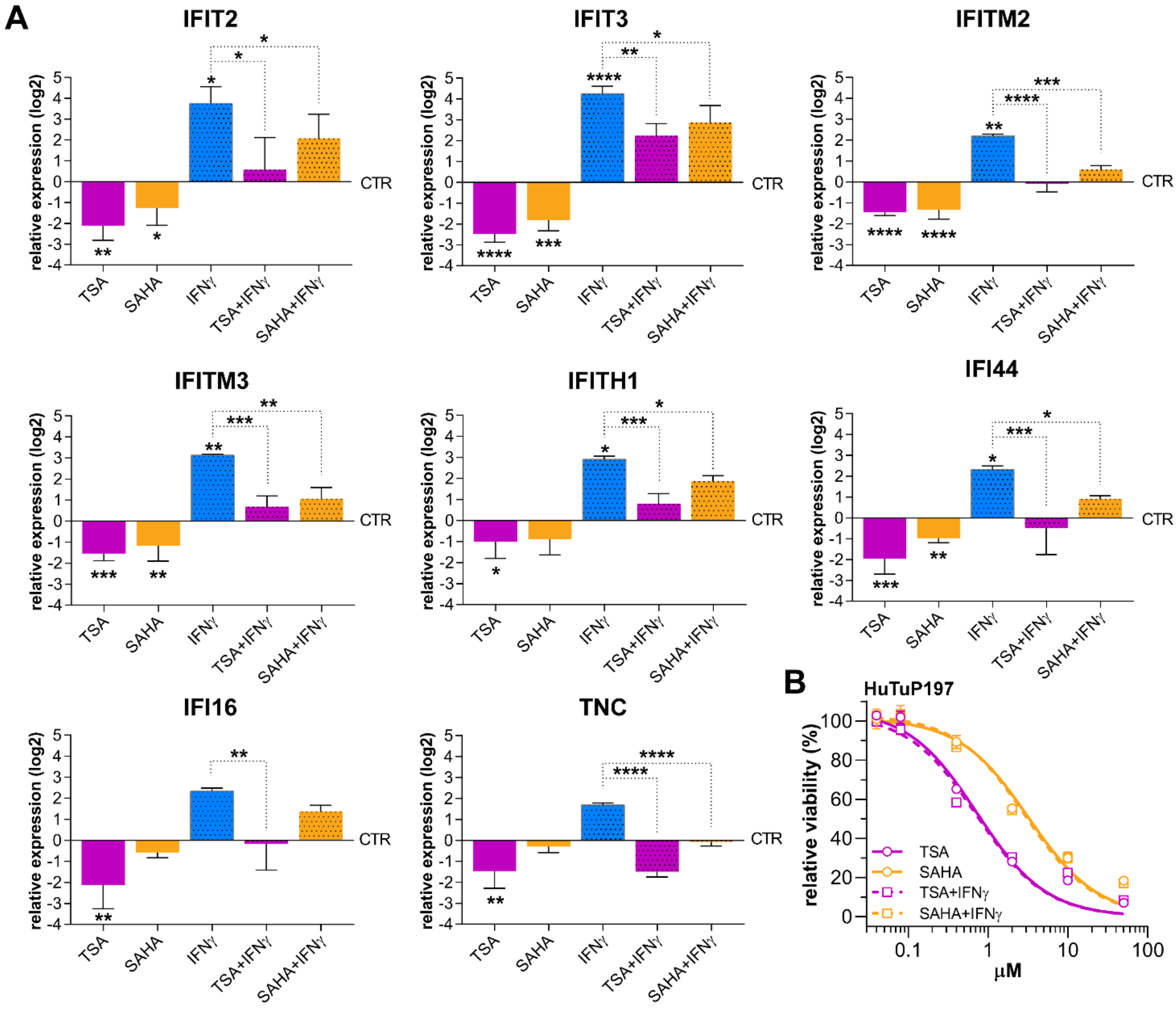

Figure S7. HDI inhibit IFN pathway target genes involved in cell motility and invasion. (**A**) Bar graphs displaying the relative expression of a series of IFN signaling target genes in response to HDI treatment (5 μM for 24 h) when combined or not with IFN-γ stimulation (1 μg/mL). (**B**) Dose-response viability curves of GBM cells (HuTuP197) exposed to scalar doses of TSA and SAHA in combination or not with 1 μg/mL of IFN-γ for 72 h. * $p < 0.05$, ** $p < 0.01$, *** $p < 0.001$, **** $p < 0.0001$ by One-way ANOVA multiple comparison test.
